# Supplementary figures and images for: Ectodomain shedding of EGFR ligands serves as an activation readout for TRP channels
Source: PLoS One. 2023 Jan 20;18(1):e0280448. doi: 10.1371/journal.pone.0280448 (PMC9858409; doi:10.1371/journal.pone.0280448)

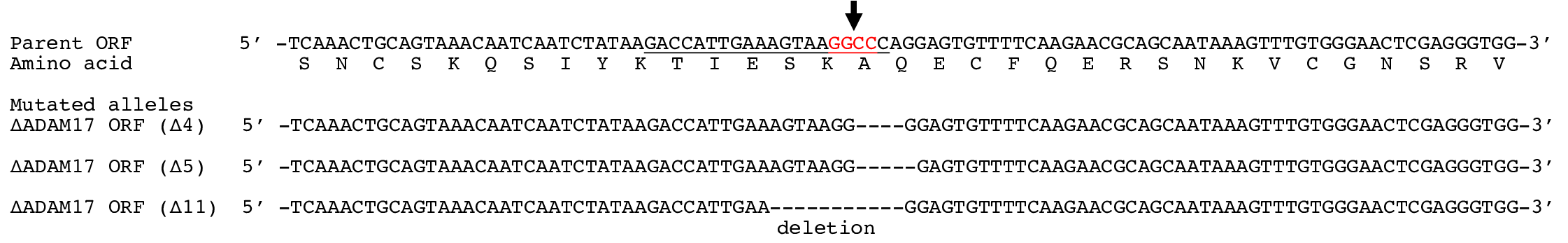

Supplement: S1 Fig — The sgRNA-target sequence is underlined. The arrow indicates a putative double-stranded break site. The restriction enzyme site (Hae III) is highlighted in red. (TIF) [file pone.0280448.s001.tif]
