# Supplementary material for: Ectodomain shedding of EGFR ligands serves as an activation readout for TRP channels
Source: PLoS One. 2023 Jan 20;18(1):e0280448. doi: 10.1371/journal.pone.0280448 (PMC9858409; doi:10.1371/journal.pone.0280448)
Supplement: Attachment — Submitted filename: Response letter.docx [file pone.0280448.s003.docx]

**Comments from the editor:**

*1. Please ensure that your manuscript meets PLOS ONE's style requirements, including those for file naming.*

We have formatted our manuscript to the PLOS ONE’s style.

*2. We note that the grant information you provided in the ‘Funding Information’ and ‘Financial Disclosure’ sections do not match.*

*When you resubmit, please ensure that you provide the correct grant numbers for the awards you received for your study in the ‘Funding Information’ section.*

We apologize that we had entered inconsistent information in ‘Financial Disclosure’. The updated information is as follows (change is highlighted in yellow).

*“A.I. was supported by Japan Society for the Promotion of Science (JSPS) KAKENHI grants 16H01377, 21H04791, 21H05113, JPJSBP120213501 and JPJSBP120218801; FOREST Program JPMJFR215T and JST Moonshot Research and Development Program JPMJMS2023 from Japan Science and Technology Agency (JST); Takeda Science Foundation; Uehara Memorial Foundation. M.T. received JSPS KAKENHI 22J10475. The funders had no role in study design, data collection and analysis, decision to publish, or preparation of the manuscript.”*

*3. We note that you have provided funding information that is not currently declared in your Funding Statement. However, funding information should not appear in the Acknowledgments section or other areas of your manuscript. We will only publish funding information present in the Funding Statement section of the online submission form. Please remove any funding-related text from the manuscript and let us know how you would like to update your Funding Statement.*

We have removed the corresponding section on funding information from the Acknowledgements Section. The correct Funding Statement is as described in the response to the point 2.

*4. In your Data Availability statement, you have not specified where the minimal data set underlying the results described in your manuscript can be found. PLOS defines a study's minimal data set as the underlying data used to reach the conclusions drawn in the manuscript and any additional data required to replicate the reported study findings in their entirety. All PLOS journals require that the minimal data set be made fully available.*

*Upon re-submitting your revised manuscript, please upload your study’s minimal underlying data set as either Supporting Information files or to a stable, public repository and include the relevant URLs, DOIs, or accession numbers within your revised cover letter. Any potentially identifying patient information must be fully anonymized.*

*We will update your Data Availability statement to reflect the information you provide in your cover letter.*

Since the source data set is only a size of an excel file, we have included it as a Supporting Information file (S1 Data). Our new Data Availability statement is as follows.

*“All data are provided in the paper and its Supporting Information file.”*

*5. Please review your reference list to ensure that it is complete and correct. If you have cited papers that have been retracted, please include the rationale for doing so in the manuscript text, or remove these references and replace them with relevant current references. Any changes to the reference list should be mentioned in the rebuttal letter that accompanies your revised manuscript. If you need to cite a retracted article, indicate the article’s retracted status in the References list and also include a citation and full reference for the retraction notice.*

After checking the references, we have verified that the reference list is complete and correct, and that there are no retracted papers in the reference list.

We also note that we have added three new papers to our list in response to the reviewers' suggestion.

**Comment from Reviewer #1**

*In this work, Tatsumi et al. developed a new molecular biology method to assay TRP channels activity exogenously expressed in cell lines, based on the use of a transforming TGF alpha shedding assay. This assay has the advantage of being low cost compared to calcium imaging or patch-clamp tecniques. That said, the information obtained by this method doesnt´t allow to monitorize TRP channel activity since the measurement is based on cummulated activity. Further, it is not useful to evaluate the activity of natively expressed TRP channels. Despite those limitations, this work might help the high-throughput screening of TRP channels ligand and to clarify the relationship between TRP channels and patho-physiologically relevant proteins as ADAM. Overall, the manuscript is well-written, well-structured, and the data support the conclusions.*

*As a main concern, authors show spontaneous TRPV1 activation (Figure 1H), however the origin of this activity remains unclear in the manuscript, since authors do not discuss this point or mention any reference supporting this. Is this TRPV1 spontaneous activation developed as a consequence of TGF alpha transfection? Has been this spontaneous activity previously reported in calcium imaging or patch-clamp experiments? If so, authors should add the corresponding references to the draft.*

We thank the Reviewer #1 for the important comments. We have now added the explanation of TRP spontaneous activity as follows (highlighted in yellow; page 9, line 202-205).

*“Finally, we evaluated the inverse agonist activity of capsazepine, which refers to the inhibitory effect of capsazepine against spontaneous TRPV1 activation. Many TRP channels exhibit spontaneous activity that is observed just by expressing them in cultured cells, which is caused by constitutive activation of upstream factors of TRP channels [29]. This spontaneous activity causes chronic pain and degeneration in nerves and other cells, making it an important therapeutic target [30,31]. We incubated TRPV1-expressing or mock-transfected cells with increasing concentrations of capsazepine for 1 h and measured the TGFα-shedding response. TRPV1-expressing cells showed a TGFα-shedding response without ligand stimulation, and the addition of capsazepine suppressed this response to a level similar to the level observed for mock-transfected cells (Fig 1H, the TGFα-shedding response is shown without subtracting the basal response).”*

**Comment from Reviewer #2**

*The present manuscript by Tatsumi and colleagues is an attempt to develop a low-cost and easy-to-access method for evaluating TRP channel activation. This will be useful for researchers, especially those who have limited access to specialized equipment, and may broaden the TRP channel field.
The present manuscript should be accepted in its present form.*

We thank the Reviewer #2 for his/her appreciation of our work.
